# Processing of Synonyms and Homographs in Bilingual and Monolingual Speakers

**DOI:** 10.5334/joc.329

**Published:** 2024-01-09

**Authors:** Clara D. Martin, Romain Pastureau, Emilia Kerr, Angela de Bruin

**Affiliations:** 1 Basque center on Cognition, Brain and Language, San Sebastian, ES; 2 Ikerbasque – Basque Foundation for Science, Bilbao, ES; 3 Universidad del País Vasco/Eusjkal Herriko Unibertsitatea, San Sebastián, ES; 4 LPL – Aix-Marseille University, Aix-en-Provence, FR; 5 Department of Psychology – University of York, York, UK

**Keywords:** Synonyms, Homographs, Double-mapping words, Bilingualism, Cross- to within-language transfer

## Abstract

Bilinguals have long-lasting experience with cross-language double-mappings (i.e., translation equivalents and interlingual homographs (or false friends)). Considering this, we examined whether bilinguals differ from monolinguals in within-language double-mapping (i.e., synonyms and homographs) processing. Across two experiments, we compared performances from Spanish monolinguals and Spanish-Basque bilinguals on a behavioral picture-word matching task. The words were all presented in Spanish, the native language of all participants. Participants responded to synonyms and homographs (both double-mappings) or single-mappings (controls). The reaction times in both experiments showed clear and significant costs in processing within-language double-mapping stimuli, as well as intrinsic differences in processing homographs versus synonyms. However, these effects did not differ between bilinguals and monolinguals. The present findings thus suggest that the bilinguals’ extensive experience with cross-linguistic double-mappings does not transfer onto within-language double-mapping processing.

## 1. General Introduction

Most languages in the world contain both single-mappings (one word referring to one concept) and double-mapping words (hereafter within-language double-mappings), commonly called synonyms and homographs. 2:1 double-mapping words, or synonyms, are two (or more) words that mean exactly or nearly the same. For instance, in English the words ‘present’ and ‘gift’ both refer to the concept of a thing given willingly to someone without payment. In contrast, homographs, 1:2 double-mapping words, refer to two (or more) words in the same language that are spelled the same way but have different meanings. For example, in English the word ‘bat’ refers to multiple concepts, namely a nocturnal flying animal and a tool to play baseball (“homographs”). While monolinguals only experience these double mappings in one language, bilinguals have double mappings (multiple words in multiple languages) for almost all concepts. The two experiments of the present project therefore aimed at exploring whether bilinguals differ from monolinguals in their processing of double-mapping words in one language because of their experience with double-mappings across languages.

Several previous studies have revealed a cost (i.e., slower and more error prone responses) in double-mapping word processing as compared to single-mapping words (Britt, Ferrara & Mirman, 2016; Rodd, Gaskell & Marslen-Wilson, 2002; Hino, Lupker & Pexman, 2002; Pecher, 2001). For instance, Rodd and colleagues (2002) showed in a series of three lexical decision experiments that homographs are recognized more slowly than single-mapping words, showing that competition between the different (unrelated) meanings of homographs slows down their processing. As for synonym processing, Britt and colleagues (2016) showed that word production in a picture-naming task was less accurate and slower for synonyms relative to single-mapping control words. This effect is explained by a slowed down lexical selection for production when there is lexical competition (i.e., various lexical entries activated by a picture/concept). This is further supported by Hino and colleagues (2002) showing that responses are also slower, in lexical decision and naming tasks, for words with many synonyms as compared to few synonyms.

Thus, double-mapping words appear to be processed differently than single-mapping words because of the competition across concepts (in the case of homographs) or lexical entries (in the case of synonyms). Importantly, however, homograph and synonym processing has been explored mainly in monolinguals so far, namely in participants who speak only one language fluently.

Yet, bilinguals are highly used to double-mappings in their daily life. This is especially true if we consider early highly-proficient bilinguals who speak two languages at a native-like level and use both languages frequently. For early highly-proficient bilinguals, most (if not all) words are double-mapping words, cross-linguistically. In fact, most concepts are linked, in the bilingual brain, to a word in their first language (L1) and another one in their second language (L2). For instance, a Spanish-English bilingual speaker has a double-mapping representation for the concept of ‘item of furniture to sleep on’: “bed” in English and “cama” in Spanish (i.e., 2 words for 1 concept; translation equivalents). Thus, most words are cross-linguistic 2:1 double-mapping words for bilingual speakers (i.e., equivalents of within-language synonyms but cross-linguistically). Furthermore, interlingual homographs – also called *false friends* – are quite common, at least in some closely-related pairs of languages. Interlingual homographs are words that correspond to different concepts in the L1 and the L2. For example, “tuna” refers to a sort of fish in English and a sort of fruit in Spanish. Thus, some words are cross-linguistic 1:2 double-mapping words for bilingual speakers (i.e., equivalents of within-language homographs but cross-linguistically).

Given the strong prevalence of cross-language double-mappings in early highly-proficient bilingual language use, the main question of the project was the following: Do bilinguals differ from monolinguals in how they process within-language double-mappings because of the prevalence of, and their experience with, cross-language double-mappings in their daily life?

Processing of interlanguage homographs (i.e., false friends) and translation equivalents have been widely studied in the bilingual lexical access/memory literature (see for instance Altarriba, 1992; Dijkstra, Grainger & Van Heuven, 1999; Gerard & Scarborough, 1989; Grainger & Frenck-Mestre, 1998; Von Studnitz & Green, 2002), but there are still only few studies on the processing of within-language homographs and synonyms in bilinguals. Only a few recent studies have compared within-language homograph processing in bilinguals and monolinguals, in a sentence context. The goal of those studies was to explore whether bilinguals differed from monolinguals in their capacity of inhibiting the inappropriate meaning of a homograph based on the sentence context (Kousaie, Laliberté, López Zunini, & Taler, 2015; Paap & Liu, 2014). In Kousaie et al. (2015) for instance, participants were presented with homographs in sentence final-position (e.g., “The doctor asked her to step onto the *scale*.”) followed by contextually-appropriate target words – *balance* – or contextually-inappropriate target words – *skin* –. Participants had to perform a sentence-target relatedness judgement. If bilinguals differed from monolinguals in inhibiting the inappropriate meaning, they would differ in performance when judging the relatedness between the sentence and the contextually-inappropriate target. Despite some group differences in electrophysiological data in Kousaie et al. (2015)’s study, neither Paap and Liu (2014) nor Kousaie et al. (2015) observed significant behavioral differences between monolinguals and bilinguals.

However, literature on the acquisition of within-language double-mapping word processing, both in terms of synonym and homograph acquisition, suggests that there might be bilingual-monolingual differences. Those learning studies usually use cross-situational statistical learning paradigms (CSSL; Smith & Yu, 2008; Yu & Smith, 2007) in which participants implicitly learn each object’s name(s) by repeatedly seeing the object in conjunction with distinct other labels. Although there are inconsistent results in the literature, some studies have shown that bilingual adults outperform monolinguals in learning novel 2:1 mappings: the learning of two different label names for the same novel object (steeper learning curve and/or greater proficiency at test; Benitez, Yurovsky & Smith, 2016; Chan & Monaghan, 2019; but see Aguasvivas & Carreiras, 2021 for no difference between bilinguals and monolinguals in similar settings).

There is also previous evidence for a bilingual advantage in learning 1:2 mappings in adults, i.e., learning to associate one novel label to two different novel objects. Poepsel and Weiss (2016) showed, in a CSSL experiment, that bilinguals learned novel 1:2 mappings faster and better than monolinguals (but see Aguasvivas & Carreiras, 2021 for no bilingual-monolingual difference).

These learning studies tend to suggest that bilingual adults benefit from faster and/or better learning of novel double-mappings, which is in line with other studies on mutual exclusivity and its flexible use in young bilingual children. For instance, Kalashnikova and colleagues (2015) showed that bilingual children tend to accept multiple-mappings (i.e., lexical overlap) to a higher level than monolingual children (Kalashnikova, Mattock, Monaghan, 2015).

To summarize, in the monolingual literature, there is previous evidence that monolinguals are slower to process existing within-language double-mapping words (i.e., synonyms and homographs) as compared to single-mapping words. In the bilingual literature, there is evidence that bilinguals outperform monolinguals in learning novel within-language double-mappings. Given that bilinguals are highly used to processing double-mappings across languages (i.e., translation equivalents and interlingual homographs), we explored whether bilinguals outperform monolinguals in the processing of existing within-language double-mappings (and not only in learning novel ones). We present two experiments, both examining how bilinguals and monolinguals process homographs and synonyms, with some differences in the material, procedure and participants. In both experiments, we expected all participants to be slower to process double-mappings as compared to single-mappings (Pecher, 2001; Rodd et al., 2002). Given the prevalence of double-mapping processing in bilinguals’ daily life, and given their outperformance in learning novel double-mappings relative to monolinguals (Benitez et al., 2016; Chan & Monaghan, 2019; Poepsel & Weiss, 2016), we expected the cost in processing double-mappings to be reduced in bilinguals as compared to monolinguals.

## 2. Experiment 1

### 2.1. Introduction

In Experiment 1, we compared monolinguals and bilinguals on a behavioral picture-word matching task. Participants were presented with one word and one picture and had to decide as fast as possible if the word and picture matched. Words were presented in Spanish, the native language of all participants. Words could be synonyms, homographs (two types of double-mappings), or single-mappings (as controls). We analyzed reaction times in the picture-word matching task for each mapping (double- versus single-mappings) and performance was compared across the two groups (monolinguals and bilinguals).

First, we expected all participants to respond more slowly to double- than single-mappings (i.e., main effect of mapping), as previously shown in monolingual populations (Britt et al., 2016; Pecher, 2001; Rodd et al., 2002). We will refer to this as the double-mapping cost. Second, our main hypothesis was that bilinguals would show a smaller double-mapping cost (i.e., smaller cost in processing double- as compared to single-mappings) as compared to monolinguals. We expected this bilingual effect to be mainly observed for 2:1 mappings (i.e., synonyms) since those are the most common double-mappings encountered by bilinguals in cross-language situations (i.e., translation equivalents exist for almost all words while interlingual homographs exist for some but not all words).

We also included presentation order in the analysis, since each item was presented twice during the experiment. Each picture related to a synonym was presented once associated to each word (e.g., picture of an olive was presented once together with the word ‘oliva’ and once with the word ‘aceituna’ [both words meaning ‘olive’ in Spanish]). Each homograph was presented once with each of its associated meanings (e.g., the word ‘banco’ [meaning ‘bench’ and ‘bank’] was presented once together with the picture of the seat and once with the picture of the financial institution). Single-mapping words (controls) were presented twice the same. We expected a significant effect of presentation order, mainly for control words, with participants being faster in the matching task during the second presentation of a same picture/word. For the double-mappings we expected participants to be faster the second time only if the first presentation of the other word/concept did not provide additional conflict at the second presentation time. Because bilinguals are highly used to double-mappings, it might be the case that they are faster than monolinguals to accept a match with the new meaning/word after having processed the other meaning/word first. In such a case, we would expect an interaction between presentation order and group.

Finally, we asked each participant, at the end of the experiment, to indicate their preference for each of the two synonyms associated to the same picture (e.g., whether they preferred “sofa” or “couch”) and to each of the two meanings associated to a homograph (e.g., whether, to them, “bat” was more strongly associated with the tool or animal). We included preference in the analysis, and expected participants to be faster to respond when presented with their preferred picture/word.

### 2.2. Methods

#### Participants

Two groups of participants were recruited from the BCBL participant database: 13 Spanish-Basque bilinguals (mean age = 25.8, *SD* = 4.68; 1 male) and 11 Spanish *functional* monolinguals (mean age = 24.2, *SD* = 4.69; 1 male).^1^ Monolingual participants were *functional* monolinguals in the sense that they did not use another language than Spanish in their everyday life. We will refer to them as “monolinguals”.

All participants were assessed on their language proficiency, use and exposure in Spanish, Basque and English. Participants were asked to rate on a scale from 0 to 100% how often they were exposed to each language, as well as to provide their age of acquisition. The objective assessment procedure was part of the BEST test (de Bruin, Carreiras & Duñabeita, 2017). Participants were tested on a picture naming task providing an objective rating of proficiency on a 0–65 scale and on the adaptation of the LexTale task to Spanish, Basque and English, providing a percentage of correct lexical decisions (see Table S1 in supplementary material).

Bilingual participants were all early bilinguals (both languages acquired before the age of 3), highly-proficient in both languages, and used both languages on a daily basis (see Table S1). Monolingual participants could only speak Spanish fluently, which was learned from birth, and their knowledge of Basque was minimal and lately acquired. Despite calling those groups monolinguals (Spanish speakers) and bilinguals (Spanish-Basque speakers), all participants knew some English, which was unavoidable given the prevalence of English learning at school. Still, the two groups were matched on age of acquisition (*p* = .98) and level in English (BEST: *p* = .06; LexTale: *p* = .35), which was kept as low as possible (low or intermediate levels; see Table S1). Some monolingual participants reported some knowledge of another language (German, French or Italian). Their exposure (3.3% *SD* = 5.8) and proficiency (4.3 *SD* = 1.5) in those languages were minimal. All participants were borne and living in the Basque Country, meaning in a bilingual environment.

All participants had at least high school education level, no neurological, psychiatric, language or hearing impairment, were right-handed and had normal or corrected to normal vision. The authors assert that all procedures contributing to this work comply with the ethical standards of the relevant national and institutional committees on human experimentation (BCBL Ethics Review Board Approval number 040419SM) and with the Helsinki Declaration of 1975, as revised in 2008. All participants gave their written consent at the beginning of the experiment.

#### Stimuli

The stimuli consisted of 60 Spanish words divided into 4 categories: 15 homographs and 15 single-mapping control words (hereafter hom-controls), 15 synonym pairs and 15 single-mapping control words (hereafter syn-controls). Words across the four categories were matched on frequency count per million (all *p* > .09), number of letters (all *p* > .33), number of syllables (all *p* > .28),^2^ imageability (all *p*>.28), familiarity (all *p* > .52; values were extracted from EsPal database, Duchon, Perea, Sebastian Galles, Martí & Carreiras, 2013). See Appendix A in supplementary material for complete word list and Table S3 for lexical characteristics of selected words. None of the selected words had a translation equivalent in Basque that was a synonym or a homograph. It was not possible to exclude cognates. However, we kept the number of Spanish-Basque cognates similar across word categories (i.e., 8 Spanish-Basque cognates among synonyms, 10 among homographs, 4 among syn-controls and 5 among hom-controls).^3^ See Appendix B in supplementary material for further information on stimulus selection and rating.

Seventy-five grey scale pictures were selected from the MultiPic database (Duñabeitia, Crepaldi, Meyer, et al., 2018) and additional web resources. Fifteen pictures depicted the meaning of the 15 synonym pairs, 30 pictures depicted the meaning of the 30 control stimuli (15 syn-controls and 15 hom-controls), and 30 pictures depicted the meanings of the 15 homographs (two meanings/pictures per word).

#### Task and procedure

Participants were presented with stimuli consisting of a picture/word pair and had to indicate by button press whether the picture and word matched in meaning. The type of presentation of the picture/word pair differed depending on the stimulus type: For synonym pairs and syn-controls, the picture was presented first (to leave time for pre-activation of the two synonyms) and followed by the word display next to it. For homographs and hom-controls, the word was presented first (to leave time for pre-activation of the two concepts) and followed by the picture display next to it. Each synonym pair was presented twice: The picture was presented once with one word and once with the other word (e.g., picture of an olive followed by the display of the word ‘oliva’, and same picture followed by the display of the word ‘aceituna’ [both words meaning ‘olive’ in Spanish]). In order to match the number of repetitions across conditions, each syn-control stimulus was also presented twice with the same picture and word repeated (e.g., picture of a bridge followed by the display of the word ‘puente’ [meaning ‘bridge’ in Spanish], with the same picture/same word display used again). Each homograph was also presented twice: One word presented with a picture for concept 1 and then the same word presented with a picture for concept 2 (e.g., word ‘cometa’ [meaning both ‘comet’ and ‘kite’ in Spanish] followed by the display of the picture of a comet, and same word ‘cometa’ followed by the display of the picture of a kite). Again, the hom-control stimuli were also presented twice (e.g., word ‘uva’ [meaning ‘grape’ in Spanish] followed by the display of the picture of a grape, with the same word/same picture display used again).

Half of the stimuli were match trials, as presented above in the examples, and the other half consisted of mismatch trials. Mismatch trials were created by associating a picture and a word from the list that had no semantic link (e.g., picture of a bridge followed by word ‘aceituna’; word ‘cometa’ followed by the picture of a grape). Note that mismatch trials were included as fillers for the purpose of the task (match/mismatch decision) and were not trials of interest. Thus, no specific matching/controlling was applied when creating mismatch pairs, and responses were not analyzed.

In total, participants were presented with 120 match and 120 mismatch trials. Among the 120 match trials, 30 were from the synonym category (15 pictures presented twice, once with each word of the synonym pair), 30 were from the syn-control category (15 pictures presented twice, associated to the same word), 30 were from the homograph category (15 words presented twice, once with each picture depicting each of the two concepts), 30 were from the hom-control category (15 words presented twice, associated to the same picture). Filler (mismatch) trials were also repeated twice and were created following the same type of display: half of them presented with the picture first and the other half with the word first.

All match and mismatch trials were presented intermixed in a pseudo-randomized order. Half of the participants were presented with synonym 1 (or concept 1) first and with synonym 2 (or concept 2) second, with this order reversed for the other half of the participants.

Each trial started with a fixation cross for 500 ms. Depending on the condition, a picture (synonym condition) or a word (homograph condition) appeared on the screen. After 1000 ms, a word (for the synonym condition) or a picture (for the homograph condition) was presented next to the item already displayed on the screen. Participants had to press one of two button keys to indicate whether picture and word matched or mismatched in meaning. Both the picture and word remained displayed until a response was made, after which the next trial began (response hands counterbalanced across participants). In case of no response, the next trial began after 3000 ms. Participants were given breaks every 60 trials and the whole experiment lasted approximately 20 minutes.

After the main task, participants were asked to perform a short rating task. First, they were asked to indicate which of the two synonyms of a pair and which of the two homographs meanings they preferred. To do so, they were presented with a picture together with the two synonym words (or with a word (homograph) together with the two pictures depicting the two meanings) and they were asked to indicate their preferred word (or picture) by button press.

#### Data analysis

The data and scripts are available on https://osf.io/q6cm3/. Reaction time (RT) data were analyzed using linear mixed-effects analyses (R version 3.6.1. lme4_1.1–21, and lmerTEST 3.1.3.). The analysis only included the picture-word match trials, fillers (mismatch trials) were excluded. The RT analysis furthermore only included correct responses. RT outliers (< 100ms or 2.5SD above/below the mean per participant and conditions mapping, type, and presentation order, using log RTs; 0.9% of correct responses) were removed too (using trimr, Grange, 2015). The first analysis included language group (monolingual = –0.5; bilingual = 0.5), mapping (single = –0.5; double = 0.5), type (synonym + controls = –0.5; homograph + controls = 0.5), and presentation (first presentation = –0.5; second presentation = 0.5), as well as their interactions, as the fixed effects. Log-transformed RTs were used as the DV (all means given in the result section are based on untransformed RTs). We started with the full model including intercepts for participants and items and all by-participant/by-item slopes. The initial model did not converge. After removing slopes explaining the least amount of variance (starting with the by-item slopes), the model converged with participant and item intercepts and all by-participant slopes apart from type and mapping × presentation.

In the second analysis we examined the role of preference (i.e., whether a participant preferred the presented word (for synonyms) or picture (for homographs)). This analysis only included double mappings (as participants could not indicate a preference for single mappings) and the fixed effects type, language group, presentation order and preference (presented word/picture is not the preferred one = 0.5; presented word/picture is the preferred one = -0.5). The model converged with participant and item intercepts and all participant slopes apart from preference × presentation and type.

### 2.3. Results

**Figure 1:**
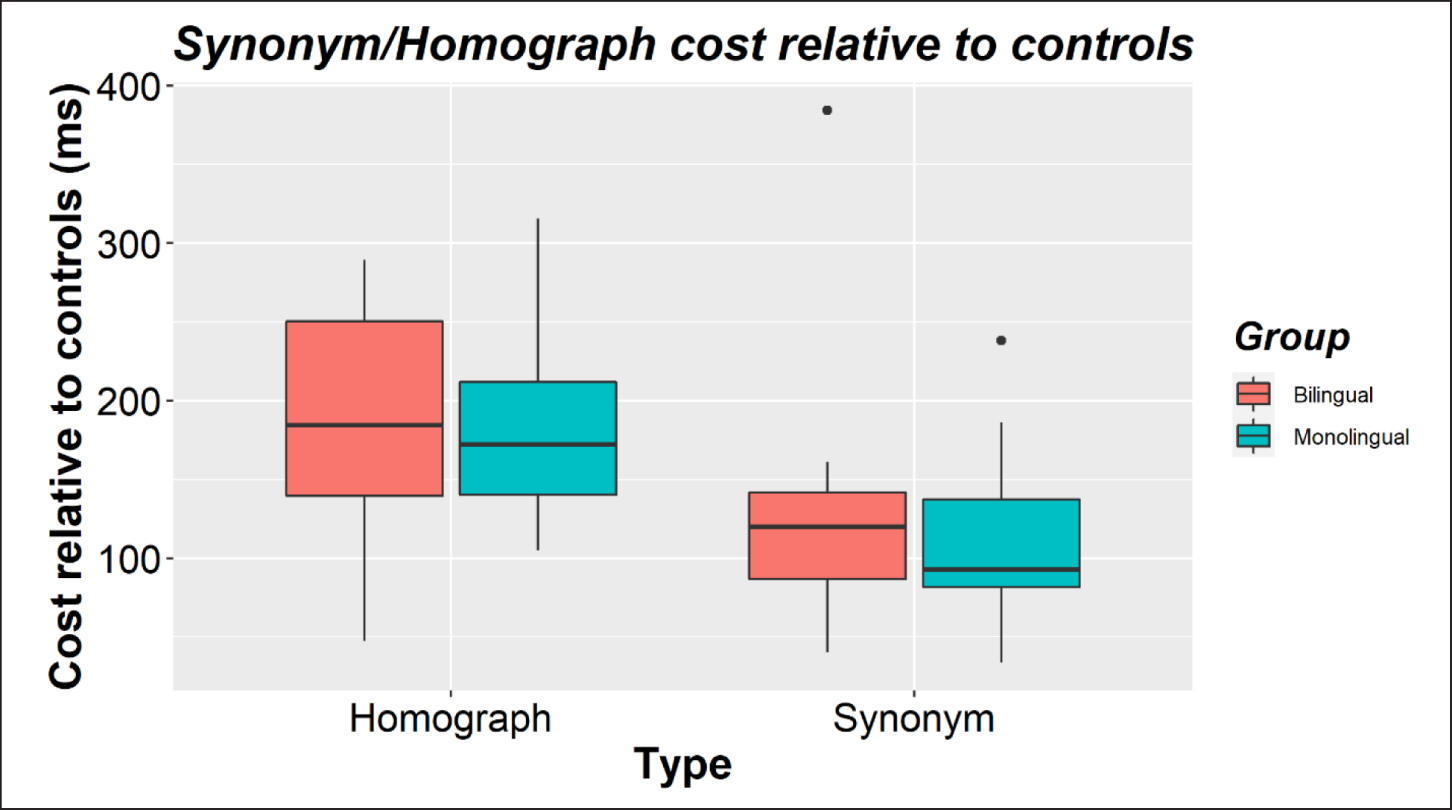
Homograph and synonym RT costs (relative to their corresponding control items) for bilinguals and monolinguals in Experiment 1. The horizontal line shows the median while black dots show outliers.

Accuracy was close to ceiling (Bilinguals *M* = 95.0%, *SD* = 2.9; Monolinguals *M* = 95.6%, *SD* = 2.1) and was not analyzed further. Table 1 shows mean RTs by condition and language group. The first RT analysis showed a main effect of mapping (β = 0.232, *SE* = 0.022, *t* = 10.465, *p* < 0.001), reflecting that people responded faster to single mappings (*M* = 559, *SD* = 104) than to double mappings (*M* = 712, *SD* = 143). There was also a main effect of type (β = 0.053, *SE* = 0.020, *t* = 2.614, *p* = 0.011), which interacted with mapping (β = 0.102, *SE* = 0.041, *t* = 2.474, *p* = 0.017). Participants showed larger double-mapping costs for homographs relative to their single-mapping controls (*M*cost = 186, *SD* = 66) than for synonyms (*M*cost = 121, *SD* = 74, see Figure 1). There was also a main effect of presentation order (β = –0.059, *SE* = 0.020, *t* = –3.003, *p* = 0.007). Participants responded faster the second time an item was presented (*M* = 612, *SD* = 113) than the first time (*M* = 652, *SD* =134). This interacted with mapping (β = 0.116, *SE* = 0.020, *t* = 5.766, *p* < 0.001). Only the single mappings benefited from being presented a second time (*M*facilitation = –73, *SD* = 51), while the double mappings did not (*M*facilitation = –3.3, *SD* = 72). There were no interactions between presentation order and type (β = 0.034, *SE* = 0.022, *t* = 1.558, *p* = 0.134) or between presentation order, mapping, and type (β = 0.036, *SE* = 0.057, *t* = 0.624, *p* = 0.539).

**Table 1:** Means (and SDs) per condition and language group in Experiment 1.


	BILINGUALS	MONOLINGUALS

*First presentation*		

Homographs	731 (201)	752 (147)

Controls homographs	596 (147)	594 (107)

Synonyms	693 (173)	685 (101)

Controls synonyms	591 (122)	603 (110)

*Second presentation*		

Homographs	760 (164)	741 (134)

Controls homographs	516 (110)	537 (86)

Synonyms	671 (173)	670 (153)

Controls synonyms	517 (103)	526 (91)


There was no main effect of group (β = –0.017, *SE* = 0.081, *t* = –0.204, *p* = 0.840). Bilinguals (*M* = 630, *SD* = 138) and monolinguals (*M* = 635, *SD* = 106) did not differ in their mean RTs. Group did not interact with mapping (β = 0.014, *SE* = 0.027, *t* = 0.520, *p* = 0.608), type (β = –0.006, *SE* = 0.020, *t* = –0.294, *p* = 0.769), or type and mapping (β = 0.002, *SE* = 0.042, *t* = 0.048, *p* = 0.963). The homograph cost (Bilingual *M* = 191, *SD* = 72; Monolingual *M* = 181, *SD* = 60) and synonym cost (Bilingual *M* = 128, *SD* = 85; Monolingual *M* = 113, *SD* = 60) did not differ between bilinguals and monolinguals (see Figure 1). Language group did not interact with presentation order either (β = 0.013, *SE* = 0.039, *t* = 0.342, *p* = 0.735, see Table 1), nor with any of the interactions with presentation order (group × mapping × presentation: β = 0.060, *SE* = 0.040, *t* = 1.503, *p* = 0.133; group × type × presentation; β = 0.004, *SE* = 0.044, *t* = 0.097, *p* = 0.924; group × mapping × type × presentation: β = 0.088, *SE* = 0.114, *t* = 0.770, *p* = 0.449).

Given that we observed no significant interaction between language group and mapping, or between language group, mapping, and type, we also conducted Bayesian analyses to assess evidence for the null effect. For each participant, we computed their homograph cost (RT difference between homographs and their control words) and their synonym cost (RT difference between synonyms and their control words). We then conducted a Bayesian Repeated Measures ANOVA using JASP 0.16.3 (keeping the default settings). We report the BF_01_ values, which quantify evidence for the null hypothesis over the alternative hypothesis. For instance, a value of 3 would indicate the data are three times more likely to be observed under the null than under the alternative hypothesis.

The best model only included type, reflecting homograph costs were larger than synonym costs. Adding Language Group to the model, to examine whether the double-mapping costs differed between bilinguals and monolinguals, showed anecdotal evidence favoring the null hypothesis of no language-group difference in double-mapping costs (BF_01_ = 2.310, error % = 1.499). To examine evidence for the interaction between language group and type, we compared the full model to a model only including the two main effects. Again, there was anecdotal evidence supporting the null hypothesis (BF_01_ = 2.756, error % = 1.533).

**Figure 2:**
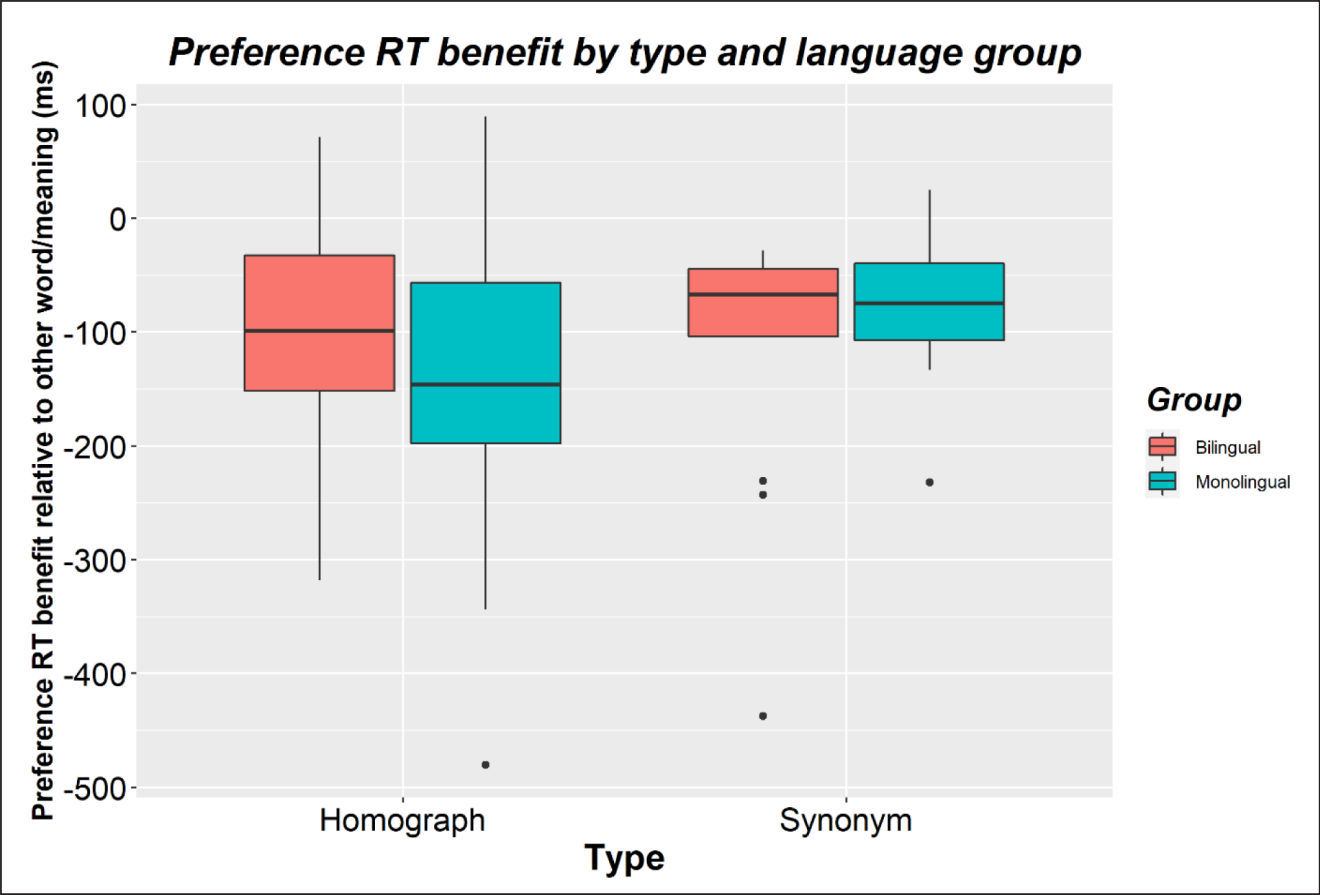
Preference benefits (i.e., faster RTs for preferred words/meanings relative to the other word/meaning) for bilinguals and monolinguals and for homographs and synonyms in Experiment 1. Negative scores reflect a benefit in processing time when seeing the preferred word or meaning. The horizontal line shows the median while black dots show outliers.

The second analysis examined the role of preference for a word within a synonym pair or a homograph’s meaning (picture). Similar to the first analysis, there was a main effect of type (*p* = 0.005) and no main effect of language group or interaction with type (all *p*s > 0.5). Given that only double-mappings were included (which, unlike single-mappings, did not differ between presentation order 1 and 2), there was no effect of presentation order (*p* = 0.840). Focusing on the role of preference, there was a main effect of preference (β = 0.139, *SE* = 0.018, *t* = 7.755, *p* < 0.001). Participants responded faster to the preferred meaning/word (*M* = 662; *SD* = 122) than to the other meaning/word (*M* = 767; *SD* = 174). This interacted with presentation order (β = -0.092, *SE* = 0.031, *t* = –2.990, *p* = 0.003). The preference benefit was larger the first time an item was presented (*Mbenefit* = –133; *SD* = 98) than the second time (*Mbenefit* = -73; *SD* = 128). Preference did not interact with type (β = 0.034, *SE* = 0.038, *t* = 0.877, *p* = 0.390) or with type and presentation (β = –0.060, *SE* = 0.071, *t* = –0.854, *p* = 0.403). The preference benefit did not differ significantly between homographs (*Mbenefit* = –120; *SD* = 134) and synonyms (*Mbenefit* = –98; *SD* = 101, see Figure 2).

Preference did not interact with language group (β = 0.001, *SE* = 0.004, *t* = 0.036, *p* = 0.972). Bilinguals (*Mbenefit* = –106; *SD* = 99) and monolinguals (*Mbenefit* = –104; *SD* = 77) showed a similar preference benefit (see Figure 2). Preference did not interact with group × type (β = –0.125, *SE* = 0.076, *t* = –1.632, *p* = 0.118), group × presentation (β = 0.073, *SE* = 0.062, *t* = 1.175, *p* = 0.240), or with group × presentation × type (β = 0.059, *SE* = 0.142, *t* = 0.417, *p* = 0.681) either.

### 2.4. Discussion

While significant double-mapping costs (slower processing when presented with synonyms or homographs relative to single-mappings) were observed, there was no difference between bilinguals and monolinguals.

The significant double-mapping cost was in line with previous studies showing a disadvantage in processing synonyms and homographs as compared to single-mapping words (Britt et al., 2016; Hino et al., 2002; Pecher, 2001; Rodd et al., 2002). This disadvantage for double-mapping word processing has been shown previously, for instance, in tasks where the participants were presented with homographs, control words and pseudowords, and had to do a lexical decision task (Klepousniotou & Baum, 2007; Rodd et al., 2002), or when presented with pictures depicting synonyms and controls, and performing a picture naming task (Britt et al., 2016). Here, we show similar disadvantages in a different type of task and paradigm, reinforcing evidence for this double-mapping cost in word processing.

This double-mapping cost also interacted with stimulus type: it was larger for homographs than for synonyms. This difference in the magnitude of the double-mapping cost between homographs and synonyms might stem from the difference in processing: competition and interference at the semantic level induced by homograph processing might be more costly in terms of processing time than competition and interference at the lexical level induced by synonym processing (see Domínguez, de Vega & Barber, 2004 for ERP differences in homograph and synonym processing in semantic priming). Such a disadvantage in processing time for homographs as compared to synonyms is also in line with behavioral data reported in Domínguez and collaborators (2004)’s study, showing a positive priming effect for synonyms but not for homographs. Note that the presentation mode differed for synonyms (picture followed by word) and homographs (word followed by picture). This difference in presentation mode was unavoidable to allow pre-activation of the two synonyms when seeing a picture and pre-activation of the two meanings when reading a word. We cannot rule out that the presentation mode might have played a role in the stimulus type effect on double-mapping cost. But importantly, their corresponding control words followed the same order. Therefore, participants did not know whether to expect single- or double-mappings for any given presentation order.

Furthermore, participants were faster to process an item when it was presented the second time, but this advantage was only present for single-mappings and not for double-mappings. This repetition advantage might be similar to the classical repetition effect: Participants are better and faster to process a stimulus when it is repeated (in word and picture identification, Durso & Johnson, 1979; Morton, 1979; lexical decision, Salasoo, Shiffrin & Feustel, 1985). Here, it is important to keep in mind that stimuli in first and second presentation were strictly identical for single-mapping (control) items: if the picture/word pair of a pineapple was presented once, the exact same pair was presented the second time. As for double-mapping items, the repetition was not strictly identical: if a picture of an olive was presented together with the word ‘aceituna’, the second presentation contained the same picture presented the first time, but now shown with the other synonym (i.e., ‘oliva’ in this example). Similarly, if the word ‘cometa’ was displayed together with the picture of a comet, the second presentation contained the same word presented at first, but associated with the picture of a kite. This is likely to explain the larger repetition advantage for single- as compared to double-mappings.

Finally, as expected, participants were faster to respond when their preferred mapping was presented. When presented with synonyms, participants were faster to respond when presented with their preferred synonym of the pair. When presented with homographs, participants were faster to respond when presented with their preferred interpretation of it. This preference benefit was larger the first time an item was presented than the second time.

These effects discussed above clearly show that synonyms and homographs were processed differently than single-mappings. However, no difference between bilinguals and monolinguals was observed. This null effect was supported by the Bayesian analysis. Although the evidence for the null was only anecdotal, if anything, any evidence for the alternative hypothesis would have been the opposite direction of our hypothesis. While we hypothesized the bilinguals’ costs to be smaller than the costs of monolinguals, the observed bilingual costs were slightly larger than the monolinguals’ costs. Still, this experiment was run with a limited number of stimuli and participants, which might be the reason no group differences were observed. Furthermore, the monolingual participants in this experiment were living in a bilingual environment, the Basque Country, a region of Spain in which most oral and written information is constantly provided in both Spanish and Basque, and in which many people learn the two languages from birth. This bilingual environment for monolinguals might have minimized the difference in behavior between the two groups. Indeed, previous studies suggest that monolinguals living in bilingual environments differ from those living in monolingual environments (Bice & Kroll, 2019), likely because of their exposition (and thus incidental learning; Saffran, Newport, Aslin, Tunick & Barrueco, 1997) to the “other” language they do not know. We therefore ran a second experiment in which we increased the number of participants and the number of stimuli, and we tested monolinguals *not* living in a bilingual region.

## 3. Experiment 2

### 3.1. Introduction

Experiment 2 was similar to Experiment 1 unless specified otherwise in the method section. The main goal and hypotheses were the same: We explored synonym and homograph processing in a picture-word matching task, comparing performance in monolinguals and bilinguals. Contrary to Experiment 1, we recruited monolinguals living in a monolingual environment, to maximize potential differences between the two populations. Consequently, we expected bilinguals to show a smaller double-mapping cost as compared to monolinguals, and we expected this bilingual effect to be mainly observed for 2:1 mappings (synonyms).

### 3.2. Methods

#### Participants

Two groups of participants were recruited from the BCBL participant database: 41 Spanish-Basque bilinguals (mean age = 26.5, *SD* = 4.78; 7 male) and 40 Spanish *functional* monolinguals (hereafter monolinguals; mean age = 26.7, *SD* = 4.96; 5 male). Given that Experiment 1 showed no difference in double-mapping costs between bilinguals and monolinguals, we could not conduct a power analysis based on previously observed effect sizes. We therefore selected our sample size following recommendations for LME analyses that recommend 1600 observations per condition for a properly powered experiment exploring reaction times in a repeated measures design (Brysbaert & Stevens, 2018). We aimed for 2000 observations per condition (40 participants and 50 trials/condition) in order to get to a minimum of 1600 after data cleaning.

All participants were assessed on their language proficiency, use and exposure in Spanish, Basque (for bilinguals) and English. Participants were asked to rate on a scale from 0 to 100% how often they were exposed to each language, as well as to provide their age of acquisition. The objective assessment procedure was part of the BEST test (de Bruin, et al., 2017). Participants were tested on a picture naming task providing an objective rating of proficiency on a 0–65 scale and on the adaptation of the LexTale task to Spanish, Basque and English, providing a percentage of correct lexical decisions (see Table S2 in supplementary material).

Bilingual participants were all early bilinguals (both languages acquired before the age of 3), highly-proficient in both languages, and used both languages on a daily basis (see Table S2). Monolingual participants could only speak Spanish fluently, which was learned from birth (see Table S2). As in Experiment 1, all participants had some knowledge of English, which is inevitable in young adults living and studying in Spain. Still, the two groups were matched on age of acquisition (*p* = .94) and level in English (BEST: *p* = .69; LexTale: *p* = .36), which was kept as low as possible (low or intermediate levels; see Table S2). Some monolingual participants reported some knowledge of French, Catalan and Galician.^4^ Their exposure (9.3% *SD* = 12.7) and proficiency (3.3 *SD* = 2.1) in those languages were minimal. Importantly, in comparison to Experiment 1, monolingual participants were all born and living in a monolingual environment, in the Murcia region in the South of Spain.

All participants had at least high school education level, no neurological, psychiatric, language or hearing impairment, were right-handed and had normal or corrected to normal vision. The authors assert that all procedures contributing to this work comply with the ethical standards of the relevant national and institutional committees on human experimentation (BCBL Ethics Review Board Approval number 191119SM) and with the Helsinki Declaration of 1975, as revised in 2008. All participants gave their written consent at the beginning of the experiment.

#### Stimuli

Stimuli consisted of 100 Spanish words divided into 4 categories: 25 homographs and 25 single-mapping control words (hereafter hom-controls), 25 synonym pairs and 25 single-mapping control words (hereafter syn-controls). Words across the four categories were matched on frequency count per million (all *p* > .07), number of letters (all *p* > .28), number of syllables (all *p* > .27), imageability (all *p* > .09), familiarity (all *p* > .48; values were extracted from EsPal database, Duchon et al., 2013). See Appendix C in supplementary material for complete word list and Table S4 for lexical characteristics of selected words. None of the selected words had a translation equivalent in Basque that was a synonym or a homograph. It was not possible to exclude cognates. However, we kept the number of Spanish-Basque cognates similar across word categories (i.e., 11 Spanish-Basque cognates among synonyms, 17 among homographs, 11 among syn-controls and 11 among hom-controls).^5^ One hundred and twenty-five grey scale pictures were selected from various web resources. Twenty-five pictures depicted the meaning of the 25 synonym pairs, 50 pictures depicted the meaning of the 50 control stimuli (25 syn-controls and 25 hom-controls), and 50 pictures depicted the meanings of the 25 homographs (two meanings/pictures per word; see Pastureau, de Bruin, Martin (in preparation) for a full description of the stimulus database). See Appendix D in supplementary material for further information on stimulus selection and rating.

#### Task and procedure

The main difference with Experiment 1 was that Experiment 2 was run online, because of the COVID pandemic. The experiment was programed with jsPsych (de Leeuw, 2015), ran on the JATOS platform (Lange, Kühn & Filevich, 2015). The task automatically appeared in full screen, and the participants were asked to avoid distractions and to pass the experiment in one go. Apart from this main difference, task and procedure were similar to Experiment 2 (see section 2.2.3) unless specified otherwise in the following.

Mismatch trials were created as in Experiment 1, but the proportion of match/mismatch trials was 66/33 instead of 50/50. In total, participants were presented with 200 match and 100 mismatch trials. Among the 200 match trials, 50 were from the synonym category (25 pictures presented twice, once with each word of the synonym pair), 50 were from the syn-control category (25 pictures presented twice, associated to the same word), 50 were from the homograph category (25 words presented twice, once with each picture depicting each of the two concepts), 50 were from the hom-control category (25 words presented twice, associated to the same picture). Filler (mismatch) trials were also repeated twice and were created following the same type of display: half of them presented with picture first and the other half with word first.

All match and mismatch trials were presented intermixed in a pseudo-randomized order. Two trials containing the same word or the same picture never appear with less than 20 trials in between (average number of trials between two trials containing the same word or the same picture: 109 *SD* = 63.5). Half of the participants were presented with synonym 1 (or concept 1) first and with synonym 2 (or concept 2) second, and conversely for the other half of the participants.

Each trial started with a fixation cross for 1500–2500 ms (jittered presentation time of 1500, 1750, 2000, 2250, 2500). Depending on the condition, a picture (synonym condition) or a word (homograph condition) appeared on the screen (words always displayed on the top and pictures on the bottom). After 1000 ms, a word (for the synonym condition) or a picture (for the homograph condition) was presented above/below the item already displayed on the screen. Participants had to press a key (A or L on a Spanish keyboard) to indicate whether picture and word matched in meaning or not. Both picture and word remained displayed until a response was made, after which the next trial began (response hands counterbalanced across participants). Participants were given breaks every 100 trials and the whole experiment lasted approximately 30 minutes.

#### Data analysis

The data and scripts are available on https://osf.io/q6cm3/. The two conducted analyses were similar to Experiment 1. The RT outlier removal procedure removed 2.5% of correct trials. The first analysis examined the fixed effects (and interactions between) language group, mapping, type, and presentation order. The model converged with participant and item intercepts and all by-participant slopes apart from the three-way interaction between type, mapping, and presentation order. The second analysis focused on word/meaning preference. The model converged with participant and item intercepts and all by-participant slopes apart from type × presentation × preference and presentation × preference.

### 3.3. Results

**Figure 3:**
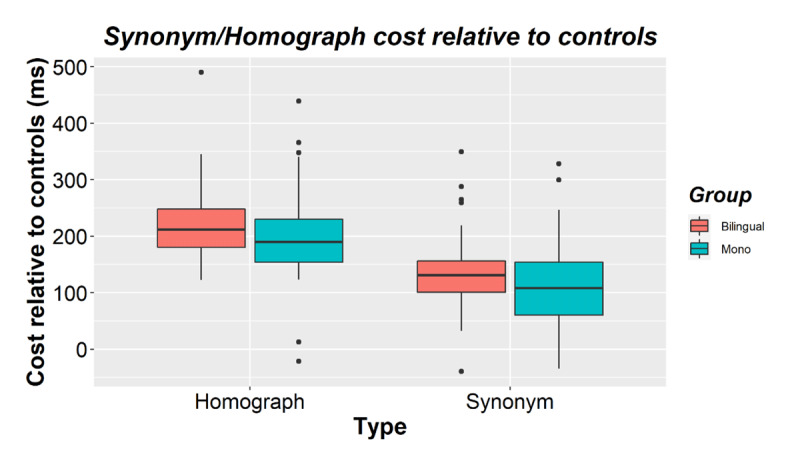
Homograph and synonym costs (relative to their corresponding control items) for bilinguals and monolinguals in Experiment 2. The horizontal line shows the median while black dots show outliers.

**Figure 4:**
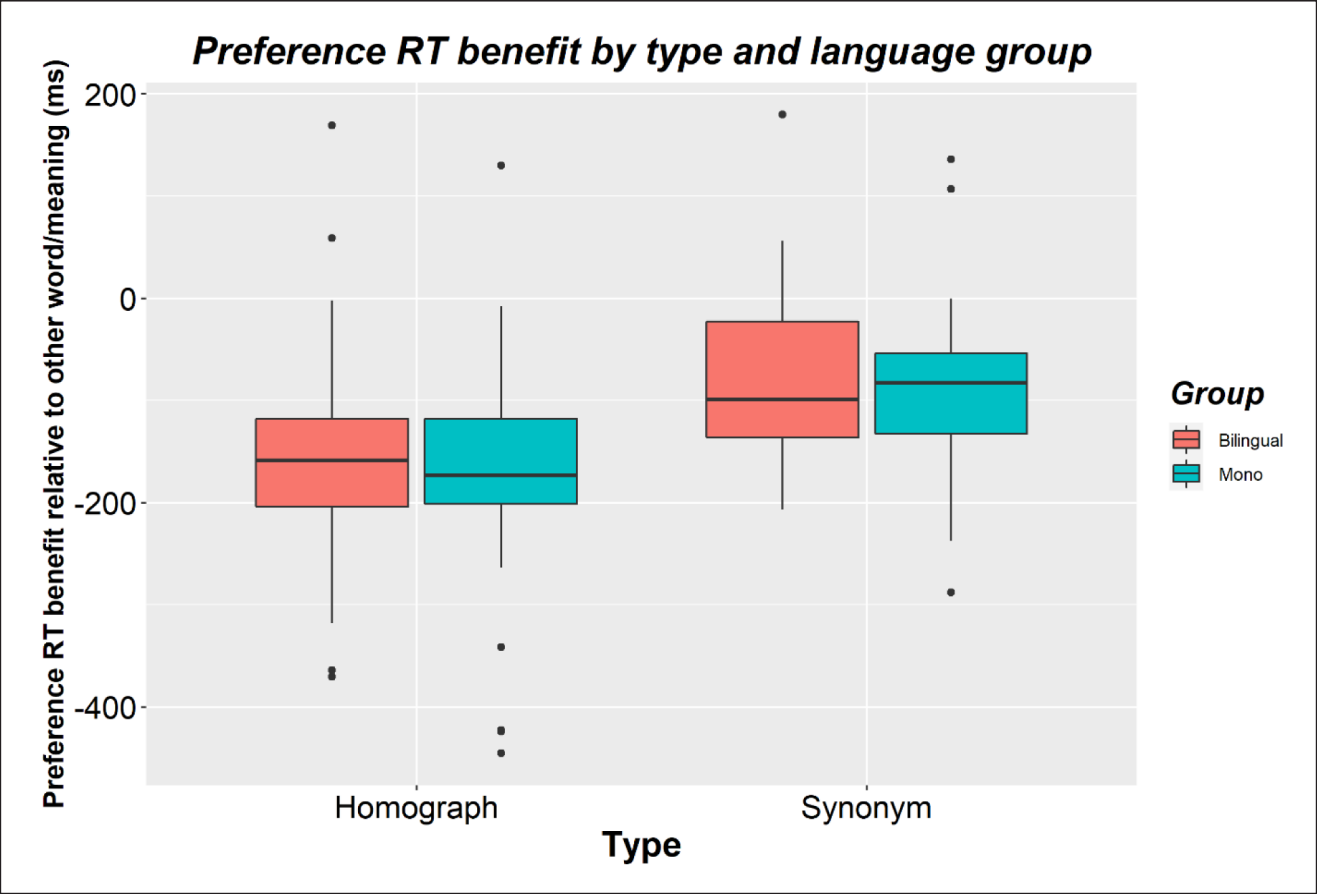
Preference benefits (i.e., faster RTs for preferred words/meanings relative to the other word/meaning) for bilinguals and monolinguals and for homographs and synonyms in Experiment 2. Negative scores reflect a benefit in processing time when seeing your preferred word or meaning. The horizontal line shows the median while black dots show outliers.

Similar to Experiment 1, accuracy was close to ceiling (Bilinguals *M* = 97.6%, *SD* = 1.6; Monolinguals *M* = 97.6%, *SD* = 1.4) and not analyzed further. The RT results are shown in Table 2 and Figures 3 and 4. There was a main effect of mapping (β = 0.230, *SE* = 0.016, *t* = 14.257, *p* < 0.001). Participants responded faster to single mappings (*M* = 653, *SD* = 173) than to double mappings (*M* = 819, *SD* = 188). There was also a main effect of type (β = 0.047, *SE* = 0.017, *t* = 2.719, *p* = 0.007), which interacted with mapping (β = 0.101, *SE* = 0.032, *t* = 3.160, *p* = 0.002). Costs were larger for homographs relative to their controls (*M*cost = 210, *SD* = 79) than for synonyms relative to their controls (*M*cost = 124, *SD* = 76, see Figure 3). There was also a main effect of presentation order (β = -0.067, *SE* = 0.009, *t* = -7.634, *p* < 0.001). Participants responded faster the second time an item was presented (*M* = 709, *SD* = 174) than the first time (*M* = 760, *SD* =189). Presentation order did not interact with type (β = -0.0005, *SE* = 0.010, *t* = –0.049, *p* = 0.961) but it did interact with mapping (β = 0.028, *SE* = 0.009, *t* = 3.074, *p* = 0.003) and with type and mapping (β = 0.093, *SE* = 0.018, *t* = 5.019, *p* < .001). All conditions were responded to fastest the second time but the homographs benefited less relative to their controls (homograph *M*benefit = –33, *SD* = 100; homograph controls *M*benefit = –63, *SD* = 87) than the synonyms (synonym *M*benefit = –68, *SD* = 82; synonym controls *M*benefit = –40, *SD* = 92).

**Table 2:** Means (and SDs) per condition and language group in Experiment 2.


	BILINGUALS	MONOLINGUALS

*First presentation*		

Homographs	855 (197)	894 (219)

Controls homographs	656 (153)	705 (191)

Synonyms	804 (192)	828 (224)

Controls synonyms	666 (178)	690 (218)

*Second presentation*		

Homographs	827 (174)	855 (200)

Controls homographs	593 (132)	641 (213)

Synonyms	741 (169)	756 (208)

Controls synonyms	611 (125)	664 (234)


There was no main effect of language group (β = –0.036, *SE* = 0.047, *t* = –0.776, *p* = 0.440). Overall RTs did not differ between bilinguals (*M* = 717, *SD* = 154) and monolinguals (*M* = 752, *SD* = 200). Group did not interact with mapping (β = 0.022, *SE* = 0.013, *t* = 1.654, *p* = 0.102), type (β = –0.025, *SE* = 0.018, *t* = -1.369, *p* = 0.175), or type and mapping (β = –0.001, *SE* = 0.024, *t* = -0.036, *p* = 0.972). Bilinguals and monolinguals did not differ significantly in their homograph cost (Bilingual *M* = 217, *SD* = 71; Monolingual *M* = 202, *SD* = 88) or in their synonym cost (Bilingual *M* = 134, *SD* = 71; Monolingual *M* = 114, *SD* = 79, see Figure 3).^6^,^7^ Language group did not interact with presentation order either (β = –0.003, *SE* = 0.018, *t* = -0.156, *p* = 0.876, see Table 2), nor with any of the interactions with presentation (group × mapping × presentation: β = 0.022, *SE* = 0.019, *t* = 1.189, *p* = 0.238; group × type × presentation; β = 0.015, *SE* = 0.019, *t* = 0.755, *p* = 0.453; group × mapping × type × presentation: β = -0.029, *SE* = 0.037, *t* = –0.776, *p* = 0.438).

Similar to Experiment 1, we also conducted a Bayesian analysis to quantify evidence for the null. The best model again only included the main effect of type, reflecting the larger homograph than synonym cost. Comparing this best model to a model also including a main effect of language group showed anecdotal evidence supporting the null hypothesis (BF_01_ = 2.331, error % = 1.416). Similarly, comparing a model with the interaction of language group x type to a model only including the main effects showed moderate evidence for the null hypothesis (BF_01_ = 4.029, error % = 3.204).

The second analysis examined the word/meaning preference.^8^ Similar to the first analysis, there was a main effect of type (*p* < 0.001), of presentation order (*p* < 0.001), and no effects of or interactions with language group (all *p*s > 0.1). There was a main effect of preference (β = 0.165, *SE* = 0.009, *t* = 19.165, *p* < 0.001). Participants responded faster to the preferred meaning/word (*M* = 761; *SD* = 191) than to the other meaning/word (*M* = 882; *SD* = 191). This interacted with presentation order (β = –0.039, *SE* = 0.013, *t* = –2.954, *p* = 0.003). The preference benefit was larger the first time an item was presented (*Mbenefit* = -135; *SD* = 101) than the second time (*Mbenefit* = –111; *SD* = 79). Preference also interacted with type (β = 0.081, *SE* = 0.014, *t* = 5.617, *p* < 0.001). The preference benefit was larger for homographs (*Mbenefit* = -163; *SD* = 106) than for synonyms (*Mbenefit* = -84; *SD* = 78, see Figure 4). This did not interact further with presentation order (β = 0.014, *SE* = 0.026, *t* = 0.547, *p* = 0.584).

Preference did not interact with group (β = –0.014, *SE* = 0.017, *t* = -0.805, *p* = 0.423). Bilinguals (*Mbenefit* = -115; *SD* = 69) and monolinguals (*Mbenefit* = –128; *SD* = 62) showed a similar preference benefit (see Figure 4). Preference did not interact with group × type (β = –0.021, *SE* = 0.029, *t* = –0.715, *p* = 0.477), group × presentation (β = –0.006, *SE* = 0.026, *t* = –0.232, *p* = 0.816), or with group × presentation × type (β = 0.067, *SE* = 0.053, *t* = 1.263, *p* = 0.207) either.

### 3.4. Discussion

Experiment 2 showed similar findings as Experiment 1, despite the changes in the stimuli used, parts of the procedure, and population. The main differences in the stimuli were that we used different words and pictures, we included 50 trials/condition (instead of 30) and the proportion of Match/Mismatch trials was 66/33 (instead of 50/50). The main differences in the procedure were that Experiment 2 was run online, and that stimuli were displayed vertically (and not horizontally) on the screen. The main differences in the populations were that we tested 40 participants in each group (instead of 13 and 11) and that the monolinguals were living in a monolingual environment. Even though a larger sample size was used and monolinguals were no longer living in a bilingual environment, no language-group differences were observed. The Bayesian analysis was consistent with the finding that double-mapping costs did not differ for bilinguals and monolinguals. Furthermore, when interpreting the anecdotal/moderate evidence for the null, similar to Experiment 1, it should be kept in mind that any evidence observed for the alternative hypothesis would have been in the opposite direction of our hypothesis.

The only key difference with Experiment 1 was that the factor Presentation interacted not only with mapping (as in Experiment 1) but also with Stimulus type. All conditions were responded to faster for the second presentation, but the homographs benefited less relative to their controls than the synonyms. Furthermore, the preference benefit was larger for homographs than synonyms, a tendency that did not reach significance but was also observed in Experiment 1.

To summarize the key findings in the synonym versus homograph matching task, homographs show a larger double-mapping cost, a smaller benefit in second presentation, and a larger preference benefit as compared to synonyms. As already suggested in the discussion of Experiment 1, those differences in processing cost between homographs and synonyms might be a consequence of interference at the semantic level (as for homographs) versus at the lexical level (as for synonyms; see Domínguez et al., 2004). We propose that because processing a homograph leads to interference at the semantic level (two possible concepts activated when processing the word), it causes larger processing costs and smaller benefits in repetition. These larger processing costs benefit significantly from a reduction of interference due to the presentation of the homograph in its preferred meanin.

## 4. General Discussion

The two experiments lead to the same observation that there is a clear cost associated with processing within-language double-mapping stimuli, but that this does not differ between bilinguals and monolinguals. Although no strong conclusions can be drawn around the absence of effects, it is important to consider that both experiments (despite using different stimuli and populations) yielded clear double-mapping effects and no group effects. Furthermore, the actual direction of the group × mapping interaction was the opposite of what was hypothesized. If anything, monolinguals tended to have *less* of a double-mapping cost relative to bilinguals (although this difference was not significant). Therefore, the results challenge the hypothesis of bilinguals having smaller within-language double-mapping costs as a consequence of their experience with double-mappings across languages.

This absence of a bilingualism effect in double-mapping processing in the L1 is consistent with previous research showing no difference between bilinguals and monolinguals in suppressing initial activation of the inappropriate meaning in sentence processing (Paap et al., 2014). Paap and collaborators (2014) showed that bilinguals did not differ from monolinguals when they had to suppress an inappropriate meaning inferred by a homograph presented in sentence-final position. Kousaie and colleagues (2015) observed subtle electrophysiological differences between monolinguals and bilinguals performing a task similar to the one used in Paap et al. (2014)’s study, but their behavioral data did not show language-group differences either. Based on those results and the present set of data, it seems that bilinguals do not differ behaviorally from monolinguals in processing double-mapping words, both when these words are presented in isolation and when they are embedded in sentences. However, further research is needed to examine whether there might be differences in the underlying neural processes in the two groups.

Another possibility is that the absence of a bilingualism effect on the within-language double-mapping cost might be due to the frequency and/or cognate status of the stimuli. Average lexical frequencies of the items were quite low, which suggests the absence of a group difference was not due to all words being processed very fast and easily by all participants (i.e., there were no ceiling effects). However, bilinguals have been found to be slower to process L1 words than monolinguals, especially low-frequent words (see for instance Gollan, Montoya, Cera & Sandoval, 2008). Furthermore, several critical items were Spanish-Basque cognates, and bilinguals can process cognates faster than non-cognates (see for instance Van Hell & Dijkstra, 2002). Importantly though, bilinguals and monolinguals did not differ in overall RTs, and not even in RTs on single-mapping (control) words (Monolinguals: *M* = 667; *SD* = 167; Bilinguals: *M* = 625; *SD* = 123; confirmed by a separate t-test just examining any potential group differences in terms of control RTs, showing *p* = .19). Thus, those potential frequency and cognate effects do not seem to have a significant impact on bilinguals when processing target and control stimuli. With critical double-mapping words matched on frequency and cognate status to their control single-mapping words, we are confident that the lack of a monolingual/bilingual difference in double-mapping cost is not due to those factors.

The absence of a bilingual effect in existing double-mapping word processing might seem at odds with similar research on novel double-mapping word learning. Several studies have shown a bilingual advantage in learning novel within-language double-mapping picture-label associations (Benitez et al., 2016; Chan & Monaghan, 2019; Poepsel & Weiss, 2016). However, not all studies have found bilingual-monolingual differences in double-mapping word learning (e.g., Aguasvivas & Carreiras, 2021). Bilingual-monolingual differences in learning might thus be limited to some specific conditions, tasks, or materials. Furthermore, such language-group difference might be specifically related to learning, with the effect disappearing progressively when item representations get consolidated and frequently used on a daily basis, as is the case for (within-language) synonyms and homographs. This assumption is consistent with a recent study highlighting differences when bilinguals and monolinguals are tested on familiar or novel words (Morini & Newman, 2020). Morini and Newman (2020) tested monolinguals and bilinguals in a word identification task in noise, with words being familiar (in task 1) or newly learned words (in task 2). Bilinguals were less accurate than monolinguals in familiar word identification in noise. However, this disadvantage disappeared when the task had to be performed on novel words. In line with this pattern, bilingualism might come with a benefit in processing newly acquired words, but not in processing familiar words. This would be consistent with bilinguals outperforming monolinguals in novel double-mapping word learning but not (anymore) in familiar within-language double-mapping words such as homographs and synonyms.

The present findings thus suggest that the bilingual’s extensive experience with cross-linguistic double-mappings does not transfer onto within-language double-mapping processing. This statement nicely aligns with previous work showing similar lack of transfer from cross- to within-language processing: In fact, Witzel (2019) showed in a recent study that the classical masked translation priming effect in bilinguals (e.g., word ‘gift’ being processed faster when preceded by a masked presentation of the prime ‘regalo’ [gift in Spanish] as compared to an unrelated prime) was not replicated when testing masked synonym priming (e.g., ‘gift’ preceded by the prime ‘present’). Our results, together with this previous finding, tend to suggest that cross-linguistic competences/effects in bilinguals do not necessarily transfer to or impact word processing within the native language. Bilinguals, when they have to recognize or produce a word following a concept display (e.g., a picture), constantly have to select the appropriate lexical item among two. For instance, in a Spanish context, the picture of a dog should lead to the selection of the word “perro” but not “txakur” [meaning “dog” in Basque]. Our main hypothesis was that if lexical selection for double-mapping words is “trained” more in bilinguals, this would percolate in a monolingual context and make them more efficient in within-language lexical selection for double-mappings, as compared to monolinguals. This hypothesis was not confirmed, suggesting that bilinguals and monolinguals behave similarly in lexical selection for double-mappings in the native language.

Still, this result is at odd with Dylman and Barry (2018)’s study revealing similarities in monolingual and bilingual lexical selection: The authors showed that, in speech production, bilinguals were faster to produce words in their second (weaker) language when primed by their translation equivalent, and similarly monolinguals were faster to produce synonymous alternative (less frequent) names when primed by the synonym most common name (Dylman & Barry, 2018). The authors concluded that there are similar facilitatory connections between translation equivalents in bilinguals and synonyms in monolinguals. Based on the present results, we argue that there might be similar facilitatory connections between translation equivalents in bilinguals and synonyms in monolinguals, but no similar facilitatory effects within bilingual participants, for both translation equivalent and synonym priming. It might also be that cross-linguistic facilitatory effects percolate to within-language processing for word production but not perception. Further studies are needed to reconcile those different aspects and apparently contradictory findings on cross- and within-language facilitation processing in bilingual speakers.

To conclude, the two experiments reported here show clear and significant costs in processing double-mapping as compared to single-mapping items, as well as intrinsic differences in processing homographs versus synonyms. However, we did not observe any significant difference in the way monolinguals and bilinguals process double-mapping items, despite their prevalence in bilinguals’ life due to the constant usage of translation equivalents for the same concept and frequent encounter of interlingual homographs. Those results do not support the hypothesis that a bilingual’s extensive experience with cross-linguistic double-mappings transfers onto within-language double-mapping processing.

## Data Accessibility Statement

The data that support the findings of this study are openly available in https://osf.io/q6cm3/. The stimuli are provided in the appendices.

## Additional Files

The additional files for this article can be found as follows:

10.5334/joc.329.s1 Supplementary Material. Tables S1 to S4.

10.5334/joc.329.s2 Appendix. Appendixes A to D.

## Footnotes

Note that the sample size was low in Experiment 1 because it was an initial experiment with limited access to monolingual participants. Experiment 2 (see below) used a larger sample size to replicate Experiment 1.

Synonyms and homographs were not matched on number of letters (*p* = .007) and number of syllables (*p* = .023). Importantly, each word category was matched with their controls on all variables.

Syn-control and hom-control were presented twice during the experiment (see Task and procedure) and this resulted in a similar number of cognate words presented in each category.

Removing these participants from the analyses did not change the results.

Syn-control and hom-control being presented twice during the experiment (see Task and procedure) this resulted in a similar amount of cognate words presented in each category.

We carried out additional analyses using percentage L1 exposure as a continuous variable instead of the categorical comparison between bilinguals and monolinguals. L1 exposure was used as a measure of how much participants used one language (in the case of monolinguals, L1 exposure was very high) or two languages (in the case of bilinguals, L1 exposure was lower). This analysis did not show any main effects of or interactions with language exposure either.

As an exploratory analysis we also examined the mismatch (filler) items to see if bilingual-monolingual differences emerge under more difficult conditions (i.e., when the picture and word mismatch). The mismatch items too showed a main effect of mapping (with longer RTs in response to double mappings) but no main effects of or interactions with language group.

As an exploratory analysis, we also examined whether preference simply reflected word frequency (e.g., more frequent words might be preferred). We only included the synonyms in this analysis (as homographs are the same word forms). While effects of frequency were found (with faster responses for more frequent words), the preference effect remained in this analysis too, suggesting preference influenced RTs over and beyond word frequency.
