# Supplementary material for: Processing of Synonyms and Homographs in Bilingual and Monolingual Speakers
Source: J Cogn. 2024 Jan 9;7(1):4. doi: 10.5334/joc.329 (PMC10785954; doi:10.5334/joc.329)
Supplement: Supplementary Material. — Tables S1 to S4. [file joc-7-1-329-s1.pdf]

Table S1: *Means (and SDs) of the subjective (Age of Acquisition and Percentage of exposure) and objective (BEST and LexTale) measures for Spanish, Basque, and English in the two language groups (Monolinguals and Bilinguals) in Experiment 1.*

**Group**	**Age of Acquisition (years)**			**Percentage of exposure**		
	Spanish	Basque	English	Spanish	Basque	English
**Monolingual**	0.0<br>(0.0)	7.4<br>(6.6)	6.3<br>(4.2)	87.2%<br>(10.0%)	8.0%<br>(8.4%)	10.0%<br>(7.1%)
**Bilingual**	0.6<br>(1.5)	0.8<br>(1.3)	6.3<br>(2.1)	50.0%<br>(17.8%)	41.5%<br>(13.5%)	6.9%<br>(4.8%)

**Group**	**BEST (percentage of correct picture naming responses)**			**LexTale (percentage of correct lexical decisions)**		
	Spanish	Basque	English	Spanish	Basque	English
**Monolingual**	99.7%<br>(0.6%)	15.7%<br>(15.0%)	47.7%<br>(14.8%)	93.9%<br>(2.8%)	58.5%<br>(9.9%)	59.2%<br>(5.7%)
**Bilingual**	99.2%<br>(1.4%)	94.0%<br>(5.7%)	56.3%<br>(7.1%)	92.8%<br>(6.6%)	89.9%<br>(6.0%)	61.8%<br>(7.4%)

Table S2: *Means (and SDs) of the subjective (Age of Acquisition and Percentage of exposure) and objective (BEST and LexTale) measures for Spanish, Basque, and English in the two language groups (Monolinguals and Bilinguals) in Experiment 2.*

**Group**	**Age of Acquisition (years)**			**Percentage of exposure**		
	Spanish	Basque	English	Spanish	Basque	English
**Monolingual**	0.6 (0.86)	N/A	6.2 (4.36)	80.9% (15.74%)	N/A	14.8% (10.43%)
**Bilingual**	0.86 (1.59)	0.9 (0.98)	6.2 (3.14)	46.6% (17.75%)	45.4% (18.23%)	8.6% (7.61%)

**Group**	**BEST (percentage of correct picture naming responses)**			**LexTale (percentage of correct lexical decisions)**		
	Spanish	Basque	English	Spanish	Basque	English
**Monolingual**	99.6% (0.87%)	1.4% (3.10%)	53.6% (11.85%)	83.3% (17.61%)	55.7% (8.38%)	66.0% (9.42%)
**Bilingual**	98.6% (2.97%)	91.2% (6.92%)	54.7% (12.40%)	85.4% (12.11%)	89.1% (6.40%)	64.1% (8.59%)

Table S3: *Means (and SDs) of lexical variables matched across conditions in Experiment 1*

	**Synonyms**	**Syn-controls**	**Homographs**	**Hom-controls**
Frequency *(count per million)*	33.6 (47.4)	18.8 (22.7)	26.3 (26.6)	11.9 (16.8)
Number of letters	6.7 (1.3)	6.3 (1.3)	5.5 (1.3)	5.4 (1.8)
Number of syllables	2.8 (0.6)	2.6 (0.6)	2.4 (0.5)	2.3 (0.8)
Imageability	6.0 (0.7)	5.7 (0.6)	5.9 (0.4)	5.9 (0.3)
Familiarity	5.9 (1.0)	5.6 (0.9)	5.8 (0.7)	5.9 (0.7)

Table S4: *Means (and SDs) of lexical variables matched across conditions in Experiment 2*

	**Synonyms**	**Syn-controls**	**Homographs**	**Hom-controls**
Frequency *(count per million)*	45.2 (88.3)	12.1 (14.6)	32.3 (32.0)	20.8 (21.3)
Number of letters	6.4 (1.6)	6.8 (1.7)	5.2 (1.2)	5.4 (1.3)
Number of syllables	2.8 (0.7)	3.0 (0.8)	2.2 (0.4)	2.4 (0.6)
Imageability	6.1 (0.5)	6.0 (0.5)	5.9 (0.4)	6.1 (0.5)
Familiarity	5.8 (0.9)	5.6 (1.1)	6.0 (0.4)	5.9 (0.6)
