# Supplementary material for: Processing of Synonyms and Homographs in Bilingual and Monolingual Speakers
Source: J Cogn. 2024 Jan 9;7(1):4. doi: 10.5334/joc.329 (PMC10785954; doi:10.5334/joc.329)
Supplement: Appendix. — Appendixes A to D. [file joc-7-1-329-s2.pdf]

Appendix A: *Stimuli used in the picture-word matching task in Experiment 1.*

Synonyms		Controls		Homographs		Controls	
Spanish	***English***	***Spanish***	***English***	***Spanish***	***English***	***Spanish***	***English***
oliva - aceituna	olive	saxofón	saxophone	tienda	shop/tent	piña	pineapple
anillo - sortija	ring	pizarra	board	flamenco	flamenco/flamingo	plancha	iron
pelota - balón	ball	calabaza	pumpkin	cometa	kite/comet	fruta	fruit
estudiante - alumno	schoolchild	dardo	dart	botones	bellboy/buttons	hipopótamo	hippopotamus
diablo - demonio	devil	hada	fairy	vela	candle/sail	bala	bullet
profesor - maestro	teacher	cazador	hunter	cola	tail/queue	flecha	arrow
colegio - escuela	school	sombra	shadow	muñeca	doll/wrist	conejo	rabbit
rincón - esquina	corner	bolsa	bag	banco	bank/bench	uva	grape
rotonda - glorieta	roundabout	cantante	singer	llama	flame/llama	guante	glove
cuaderno - libreta	notebook	puente	bridge	hoja	leaf/paper	trébol	clover
cara - rostro	face	lluvia	rain	arco	arch/bow	bellota	acorn
collar - colgante	necklace	tobillo	ankle	ladrón	thief/multiple socket	búho	owl
barbilla - mentón	chin	puño	fist	pañuelo	tissue/scarf	dedo	finger
armario - ropero	wardrobe	calamar	squid	mango	mango/handle	raíz	root
pelo - cabello	hair	manzana	apple	órgano	organ	nariz	nose

Appendix B: *Stimuli selection and rating for Experiment 1.*

To select the synonym pairs, we excluded pairs coming from regional variations (e.g., 'cacahuete' (Spain) – 'mani' (Latin America), [peanut] in English) and gender variations (e.g., 'mago' (male) – 'maga' (female), [magician] in English). We excluded synonym pairs referring to slightly different concepts (e.g., 'balcon' – 'terraza', [balcony] – [terrace]; 'diente' – 'muela', [tooth] – [molar]) and very low-frequent or archaic words (e.g., 'delantal' – 'mandil', [apron]). As for homographs, we avoided archaic or very low-frequent words. This led to a pre-selection of 30 synonym pairs and 24 homographs. Sixty control (single-mapping) words were extracted from the same database for further rating.

Eight Spanish native speakers were asked to rate the pre-selected stimuli on the following criteria. (1) Name agreement: How well does the picture represent the word? (On a scale from 1 to 7 where 1 meant 'very badly' and 7 meant 'very well'); (2) Synonym pair similarity: How well can be the two words represented by one same picture? (On a scale from 1 to 7 where 1 meant 'not at all' and 7 meant 'perfectly').

We selected only the items that received a minimum average of 5 out of 7 on name agreement. As a result, we selected 15 synonym pairs and 15 syn-controls, and 15 homographs and 15 hom-controls. The mean name agreement was 6.27 (*SD*=.57) for the selected synonyms, 6.36 (*SD*=.47) for the selected homographs, 6.58 (*SD*=.43) for the selected syn-control stimuli and 6.67 (*SD*=.44) for the selected hom-control stimuli. The mean synonym pair similarity was 6.41 out of 7 (*SD*=0.45), showing that the synonym pairs were indeed very close in meanings.

Each participant in the main task was also asked, at the end of the experiment, to assess name agreement and synonym similarity. The results of this post-test showed that participants themselves also considered the synonyms as very similar in meaning (5.42 out of 7 (*SD*=1.63)) and considered the word/picture associations to have a high name agreement (6.64 out of 7 (*SD*=.82)).

Appendix C: *Stimuli used in the picture-word matching task in Experiment 2.*

Synonyms		Controls		Homographs		Controls	
Spanish	***English***	***Spanish***	***English***	***Spanish***	***English***	***Spanish***	***English***
oliva - aceituna	olive	hada	fairy	raíz	plant root/square root	oso	bear
hoyo - agujero	hole	lupa	magnifier	gato	cat/car jack	toro	bull
alumno - estudiante	student	árbol	tree	arco	bow/arch	miel	honey
coche - automóvil	car	araña	spider	hoja	leaf/sheet	bebé	baby
ave - pájaro	bird	robot	robot	vela	candle/sail	bala	bullet
balón - pelota	ball	brazo	arm	mono	monkey/overalls	taza	mug
banana - plátano	banana	esponja	sponge	polo	stick/card symbol	ceja	eyebrow
mentón - barbilla	chin	charco	puddle	raya	line/ray (fish)	león	lion
guerra - batalla	war	guante	glove	goma	eraser/rubber band	pizza	pizza
ebrio - borracho	drunk	escoba	broom	cubo	cube/bucket	leche	milk
pelo - cabello	hair	ventana	window	banco	bench/bank	reloj	watch
camino - sendero	pathway	cerebro	brain	radio	device/radius	cerdo	pig
cara - rostro	face	botella	bottle	llama	flame/llama	mosca	fly
casa - hogar	house	ajedrez	chess	metro	subway/measuring tape	lápiz	pencil
escuela - colegio	school	pizarra	chalkboard	pluma	feather/pen	águila	eagle
collar - colgante	necklace	saxofón	saxophone	mango	fruit/handle	conejo	rabbit
libreta - cuaderno	notebook	tijeras	scissors	planta	plant/sole of the foot	lluvia	rain
flaco - delgado	skinny	bombero	firefighter	tienda	shop/tent	vestido	dress
diablo - demonio	demon	sombrero	hat	ladrón	thief/multiple plug	cerveza	beer
nevera - frigorífico	fridge	desayuno	breakfast	muñeca	wrist/doll	hormiga	ant
rotonda - glorieta	roundabout	paraguas	umbrella	trompa	elephant/French horn	abanico	fan
gordo - obeso	fat	acordeón	accordion	granada	pomegranate/hand grenade	cuchara	spoon
maestro - profesor	teacher	submarino	submarine	pañuelo	tissue/scarf	ombligo	belly button
pez - pescado	fish	termómetro	thermometer	botones	buttons/bellboy	piano	piano
pistola - revólver	hand gun	invernadero	greenhouse	flamenco	flamenco (dance)/flamingo	guitarra	guitar

Appendix D: *Stimulus selection and rating for Experiment 2.*

Stimulus pre-selection was performed similarly to Experiment 1 (no regional or gender variations etc. see Appendix B). This led to a pre-selection of 54 synonym pairs, 55 homographs and 100 controls.

Twenty Spanish native speakers took part in a rating task. They were asked to do (1) a picture naming task: type the name(s) of the picture displayed on the screen; (2) a definition task: type the definition(s) of the word displayed on the screen; (3) a picture-word pair agreement task: rate on a 1-5 scale how much the picture and the word displayed on the screen are not related at all ('1') or correspond perfectly ('5'); (4) a word-word matching task for synonyms only: rate on a 1-5 scale how much the two words displayed on the screen are not related at all ('1') or completely similar ('5'); (5) a picture-word preference task for synonyms only: choose the synonym that describes the best the picture, and rate on a 1-5 scale your preference between no preference at all ('1') and strong preference ('5'); (6) a word-picture preference task for homographs only: choose the picture that best represents the word, and rate on a 1-5 scale your preference between no preference at all ('1') and strong preference ('5').

For homographs and synonyms, we selected only the items that obtained a minimum of 25% occurrences in the picture naming task for both pictures (or words), and a minimum of 25% occurrences in the definition task for both definitions (or pictures). For instance, for the picture of an olive to be included, the names 'aceituna' and 'oliva' had to be proposed by at least 25% of the participants. For the word 'cometa' to be included, both its definitions (toy and piece of stone) had to be proposed by at least 25% of the participants. For those selected words, there was no other word or definition proposed by raters in the two first places, and picture-word pairs were graded with more than 4/5 for both pairs (Mean=4.8 *SD*=.23).

As a result, we selected 25 synonym pairs and 25 syn-controls, and 25 homographs and 25 hom-controls. The mean name agreement (based on the picture naming task) was 66.7% (*SD*=26.1%) for synonyms, 83.5% (*SD*=17.1%) for homographs, 96.8% (*SD*=5.3%) for Syn-control stimuli, 98.0% (*SD*=3.7%) for Hom-control stimuli. Syn-control and Hom-control stimuli were matched on name agreement (p=.38). The mean synonym pair similarity was 4.6 out of 5 (*SD*=.23), showing that the synonym pairs were indeed very close in meanings. Syn-control and hom-control stimuli were considered proper controls (i.e., single-mapping words) since the word of interest (syn-control) was the first one to be proposed for the presented picture in 93.5% (*SD*=7.7%) of the participants, and the meaning of interest (hom-control) was the first one to be proposed for the presented word in 97.5% (*SD*=4.3%) of the participants.
